# Small RNA Landscape of Platelet Dust: Platelet-Derived Extracellular Vesicles from Patients with Non-Small-Cell Lung Cancer

**DOI:** 10.3390/ncrna11030038

**Published:** 2025-05-07

**Authors:** Mafalda Antunes-Ferreira, Ilias Glogovitis, Diogo Fortunato, Silvia D’Ambrosi, Mariona Colom Saborit, Galina Yahubyan, Vesselin Baev, Michael Hackenberg, Natasa Zarovni, Thomas Wurdinger, Danijela Koppers-Lalic

**Affiliations:** 1 Cancer Center Amsterdam, Amsterdam University Medical Centers, 1081 HV Amsterdam, The Netherlands; 2 Department of Molecular Biology, University of Plovdiv, Tzar Assen 24, 4000 Plovdiv, Bulgaria; 3 Exosomics SpA, 53100 Siena, Italy; 4 Genetics Department, Faculty of Science, Universidad de Granada, Campus de Fuentenueva s/n, 18071 Granada, Spain; 5 Bioinformatics Laboratory, Biomedical Research Centre (CIBM), Biotechnology Institute, PTS, 18100 Granada, Spain; 6 Excellence Research Unit “Modeling Nature” (MNat), University of Granada, 18100 Granada, Spain; 7 Instituto de Investigación Biosanitaria ibs.GRANADA, University Hospitals of Granada, University of Granada, Conocimiento s/n, 18100 Granada, Spain; 8 RoseBio Srl, 25124 Brescia, Italy; 9 Leiden University Medical Center, Mathematical Institute, Leiden University, 2333 CA Leiden, The Netherlands

**Keywords:** plasma vesicles, platelet-derived vesicles, platelet dust, extracellular vesicles (EVs), small RNA sequencing, non-small-cell lung cancer (NSCLC), microRNA, immunocapture

## Abstract

**Background:** Platelet-derived Extracellular Vesicles, or “Platelet Dust” (PD), are reported as the most-abundant extracellular vesicles in plasma. However, the PD molecular content, especially the small RNA profile, is still poorly characterized. This study aims to characterize PD and other extracellular vesicles (EVs) in patients with non-small-cell lung cancer (NSCLC), focusing on their small RNA signatures and diagnostic potential. **Methods:** The EVs were isolated directly from the plasma of healthy donors and patients with NSCLC using the surface markers CD9, CD63, CD81 (overall EVs), and CD61 (PD). Small RNA sequencing was then performed to comprehensively profile the miRNAs. **Results:** Our analysis revealed distinct small RNA profiles in the EVs and the PD from the patients with NSCLC. The EVs (CD9-, CD63-, and CD81-positive) showed the enrichment of four miRNAs and the depletion of ten miRNAs, while the PD (CD61-positive) exhibited a more complex profile, with nineteen miRNAs enriched and nine miRNAs depleted in the patients with NSCLC compared to those of the healthy controls. **Conclusions:** This exploratory study enhances our understanding of miRNA composition within different plasma vesicle populations, shedding light on the biology of plasma vesicles and their contents. Furthermore, utilizing an extracellular vesicle isolation method with potential clinical applicability offers the prospect of improved cancer characterization and detection by selecting the most informative subpopulation of plasma vesicles.

## 1. Introduction

Extracellular vesicles have been extensively studied, but the previous investigations have primarily employed bulk isolation techniques, often neglecting the in-depth analysis of distinct vesicle populations and their origins. Among these vesicles, Platelet-derived Extracellular Vesicles, generally referred to as Platelet Dust (PD), represent the most predominant extracellular vesicle population in plasma, representing more than half of all plasma vesicles (70–90% in healthy individuals) [1]. To better understand their potential and biological role, it is crucial to further describe this population in the context of cancer, specifically in patients with non-small-cell lung cancer (NSCLC).

Extracellular vesicles contain several types of small RNA, including microRNAs (miRNAs), which have emerged as a promising new tool in the diagnosis and management of lung cancer. These small, stable RNA molecules are released by platelets into the bloodstream, and their profiles can reflect disease states, making them valuable minimally invasive biomarkers. Patients with lung cancer exhibit distinct changes in extracellular vesicles’ RNAs, which may correlate with tumor behavior, their response to therapy, and prognosis. These findings highlight their significant potential as biomarkers for managing such patients [2,3] and emphasize the need to further explore the biological relevance and distribution of various RNA types across different plasma vesicle populations.

MicroRNAs (miRNAs) are small, single-stranded, non-coding RNAs of around 22 nucleotides in length. miRNAs are important negative regulators of gene expression, post-transcriptionally impairing either mRNA stability or the translation of the target gene [4,5]. MicroRNA databases such as miRBase and MirGeneDB usually host the pre-microRNA and mature sequences, i.e., generally the most frequent molecule observed in sequencing experiments. However, it is generally accepted that sequence variants called isomiRs exist; they are not merely random technical artifacts, but the products of a regulated process [6,7,8]. Similar to canonical miRNAs, the expression profiles of isomiRs can vary during the individual development of a healthy organism and their qualitative and quantitative profiles can be greatly altered in diseases. IsomiRs can distinguish tumor cells from normal cells and discriminate different types of cancer and their subtypes [9,10].

In this study, we aim to characterize the small RNA landscape within the plasma extracellular vesicles obtained from both healthy donors and patients with NSCLC. We have conducted the extensive analysis of plasma vesicles immunocaptured using magnetic beads targeting EV-associated surface markers: CD9, CD63, or CD81 (overall extracellular vesicles, EVs) and CD61 (Platelet Dust, PD) [11]. Subsequently, total RNA was extracted from these isolated vesicles, and small RNA sequencing (small RNA-Seq) was performed. Our investigation focused on optimizing this method and characterizing small RNAs, including canonical and isoform microRNAs (miRNAs) (Supplementary Figure S1), as well as transfer RNAs (tRNAs), within both the EV and PD populations.

This exploratory study advances our understanding of miRNA composition within different plasma vesicle populations, shedding light on the biology of plasma vesicles and their contents. The different types of RNA have been identified, including templated and non-templated miRNAs and tRNAs. Additionally, the utilization of an optimized immunocapturing method for extracellular vesicle isolation directly from plasma samples offers great potential for clinical translation into enhanced cancer characterization and detection by selecting the most informative population of plasma vesicles.

## 2. Results

### 2.1. Protocol and Quality Control

**Figure 1:**
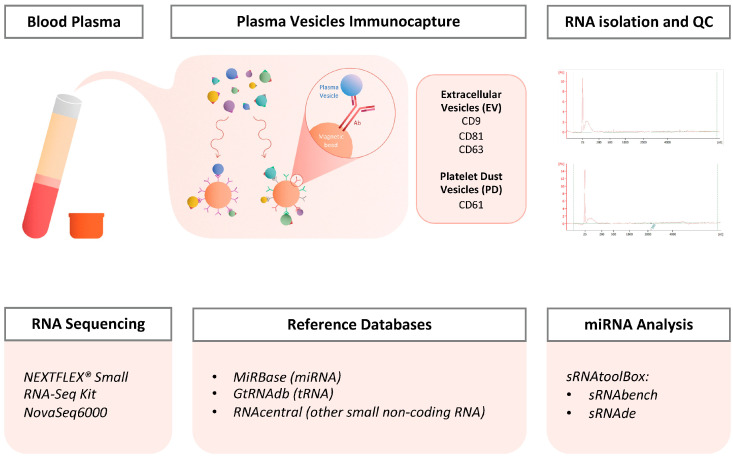
Workflow including wet- and dry-lab protocols.

Plasma was collected and processed using the standard protocol to obtain platelet-poor plasma [12], followed by the isolation of plasma vesicles via immunocapture (Figure 1). As previously described by Fortunato et al. (2022) [11], MACS beads can efficiently capture subpopulations of vesicles from plasma based on their surface phenotypes. The RNA isolated from these vesicles was evaluated using a bioanalyzer, revealing the presence of small RNAs in the samples (Figure 1). The RNA content in EVs, particularly when isolated from blood samples, is very low and often not detectable, even with the most sensitive methods available (Agilent RNA 600 Pico Kit). A peak was detected in the range of small RNA; however, accurate quantification was not possible due to the low RNA quantity present. After sequencing, several types of small RNA were mapped in our samples, with the majority consisting of miRNAs/isomiRs (~66–82%), followed by tRNAs (~10–18%) and piwiRNAs (~3–4%) (Supplementary Figure S1).

### 2.2. Characterization of Small RNA Content of Vesicles Immunocaptured from Plasma

**Figure 2:**
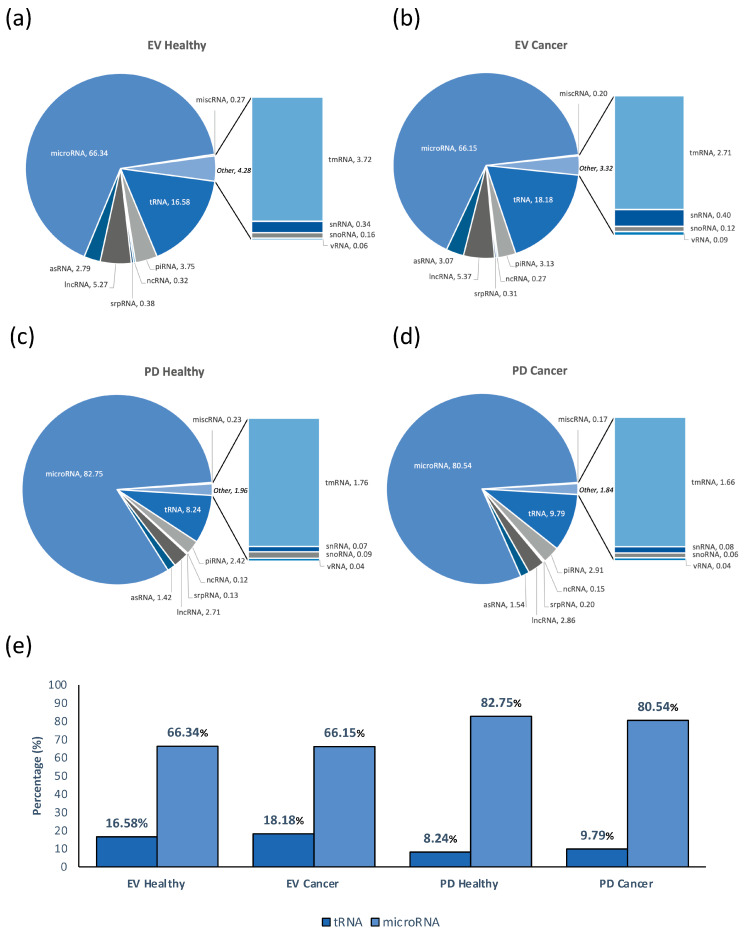
Small RNA sequencing results overview of distribution of mapped reads (percentage) between different total RNA classes (**a**–**d**), and specifically for tRNA and microRNA classes (**e**).

Our analysis showed a similar distribution of RNA classes between the healthy individuals and those with cancer (Figure 2a–d). The biggest populations presented after the removal of rRNA were microRNAs and tRNA. Noteworthy, the relative contents of microRNA and tRNA were inversely shifted in the two studied vesicle populations. The PD population contained more microRNAs than EVs, 80.54–82.75% and 66.15–66.34%, respectively. The opposite has been verified with the relative amount of tRNA, with overall EVs being enriched in tRNA with respect to PD, 16.58–18.18% and 8.24–9.79%, respectively (Figure 2e).

### 2.3. miRNA Distribution of Mapped Reads Between Different RNA Classes: Canonical, Templated and Non-Templated

**Figure 3:**
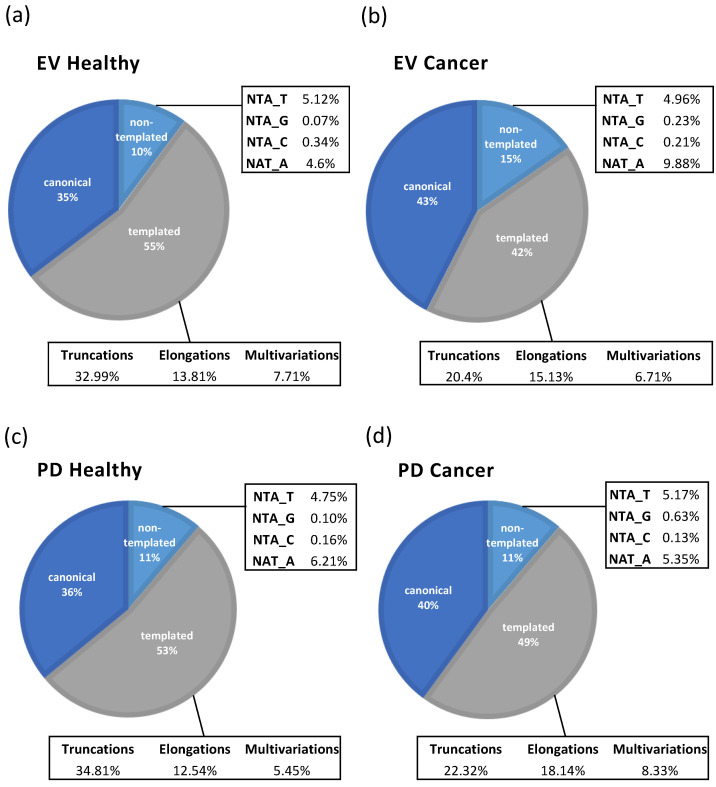
Small RNA sequencing results overview of distribution of mapped reads between different miRNA classes: canonical, templated (truncations, elongations, and multivariations), and non-templated (NTA U, G, C, and A) in extracellular vesicles (EV) from healthy (**a**), EV from patients with NSCLC (**b**), platelet-derived vesicles (Platelet Dust, PD) from healthy (**c**) and PD from patients with NSCLC samples (**d**).

The further analysis of the small RNA sequencing results allowed us to have an overview of the distribution of mapped reads between the different miRNA classes—canonical, templated (truncations, elongations, and multivariations), and non-templated (NTA T, G, C, and A) (Supplementary Figure S1)—and to assess the representativity of these different classes of miRNA in each subpopulation of plasma vesicles (Figure 3).

In the healthy samples, in the EV isolates, we observed a distribution of 35% canonical, 10% non-templated, and 55% templated miRNAs (Figure 3a), while the distribution in the PD was 36% canonical, 11% non-templated, and 53% templated RNAs (Figure 3c).

Regarding the cancer samples, in the EVs, we observed a distribution of 43% canonical, 15% non-templated, and 42% templated miRNAs (Figure 3b), and a distribution of 40% canonical, 11% non-templated, and 49% templated RNAs in the PD (Figure 3d).

**Figure 4:**
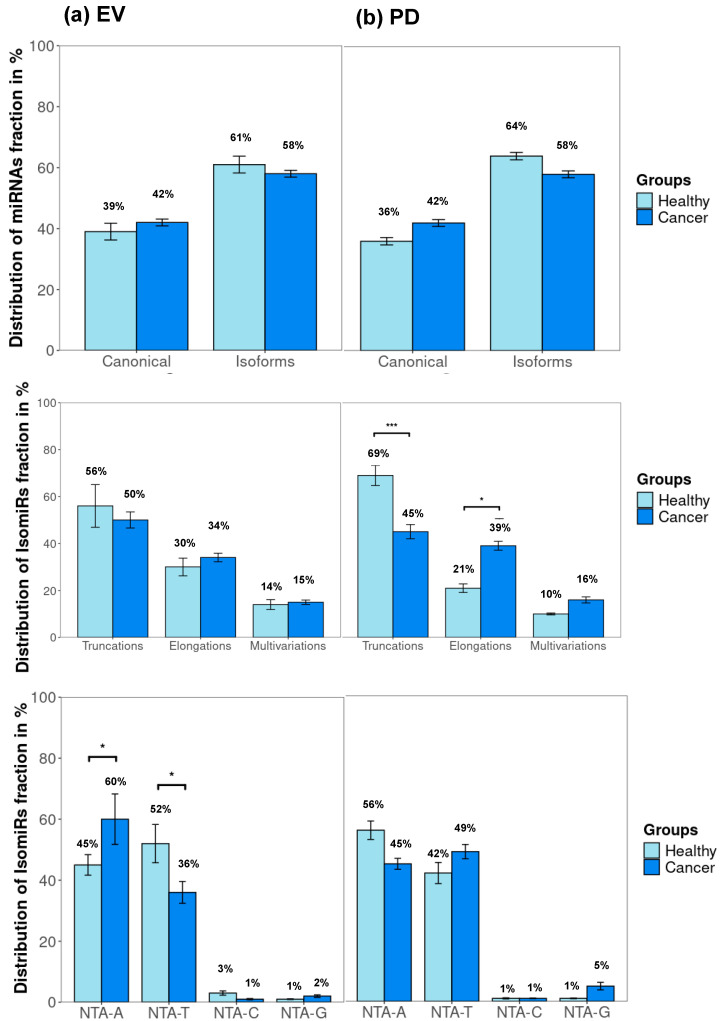

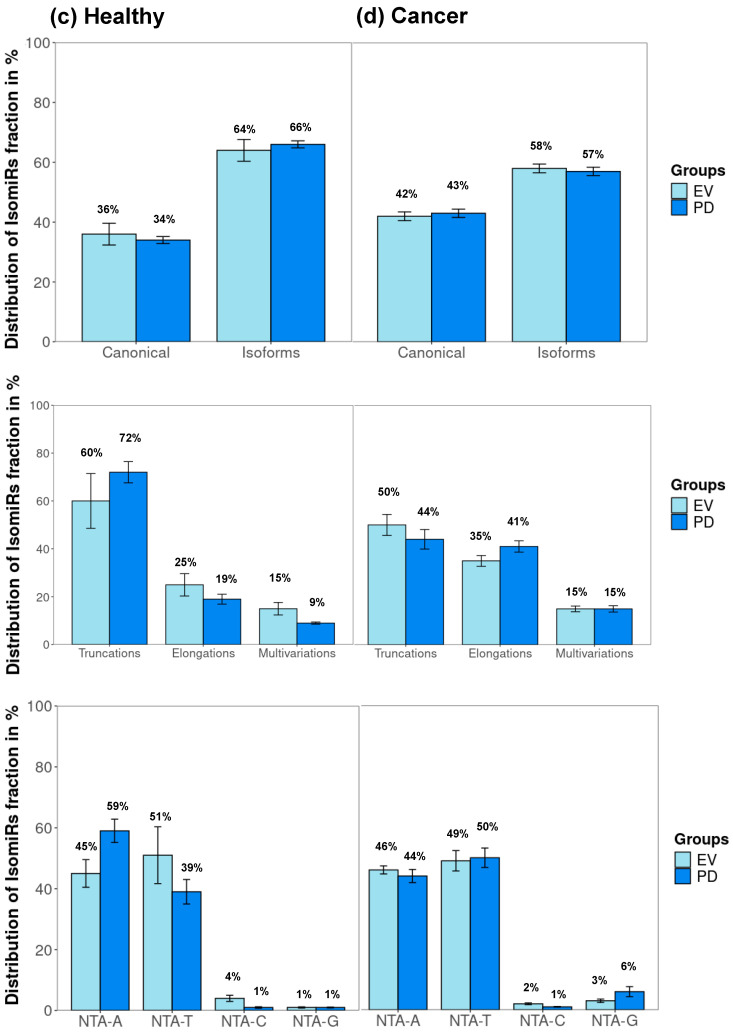
(**a**,**b**) Distributions in EVs (**a**) and platelet-derived vesicles (PD) samples (**b**) of mature miRNAs and their NTA-modified or unmodified isoforms, compared between patients with non-small-cell lung cancer and healthy donors. Error bars represent the standard deviation (SD) of mean per group, * *p* < 0.05, *** *p* < 0.001 (chi-square *t*-test). (**c**,**d**) Distributions in healthy donors (**c**) and patients with non-small-cell lung cancer (**d**) of mature miRNAs and their NTA-modified or unmodified isoforms, compared between EVs and platelet-derived vesicles (PD) samples. Error bars represent the standard deviation (SD) of mean per group.

Comparing the EV and PD vesicles between the healthy and cancer samples, it was possible to observe the enrichment of specific populations (Figure 4a,b). Interestingly, the percentage of truncations in the PD is higher in the healthy subjects, while elongations were enriched in the cancer samples (Figure 4b). The most common non-templated miRNAs classes in all the vesicles were NTA-A and NTA-T. In the two types of vesicles, PD and EVs, the presence of these miRNAs are complementary. The enrichment of NTA-A and a decrease in NTA-T in the EVs of the cancer samples was observed compared with those of the healthy samples. In the PD, the inverted trend was observed, showing a decrease in NTA-A and an increase in NTA-T in the EVs from the cancer samples (Figure 4a,b).

Comparing the two populations of vesicles, the EVs and the PD, we observed a similar percentage of canonical and isoform miRNAs, but when exploring further within the templated and non-templated isoforms, some sub-trends were observed. The PD population from the healthy samples is enriched with truncations and depleted of elongations and multivariations (Figure 4c). In the cancer samples, the PD population is depleted of truncations and enriched with elongations (Figure 4d). In the healthy samples, some further variation was observed, with PD being enriched in NTA-A, and EVs being enriched in NTA-T (Figure 4c). In the cancer samples, no major differences were observed between the EVs and the PD (Figure 4d).

### 2.4. Differences Between Healthy and Cancer miRNAs/isomiRs in the EVs and in the PD Populations

**Figure 5:**
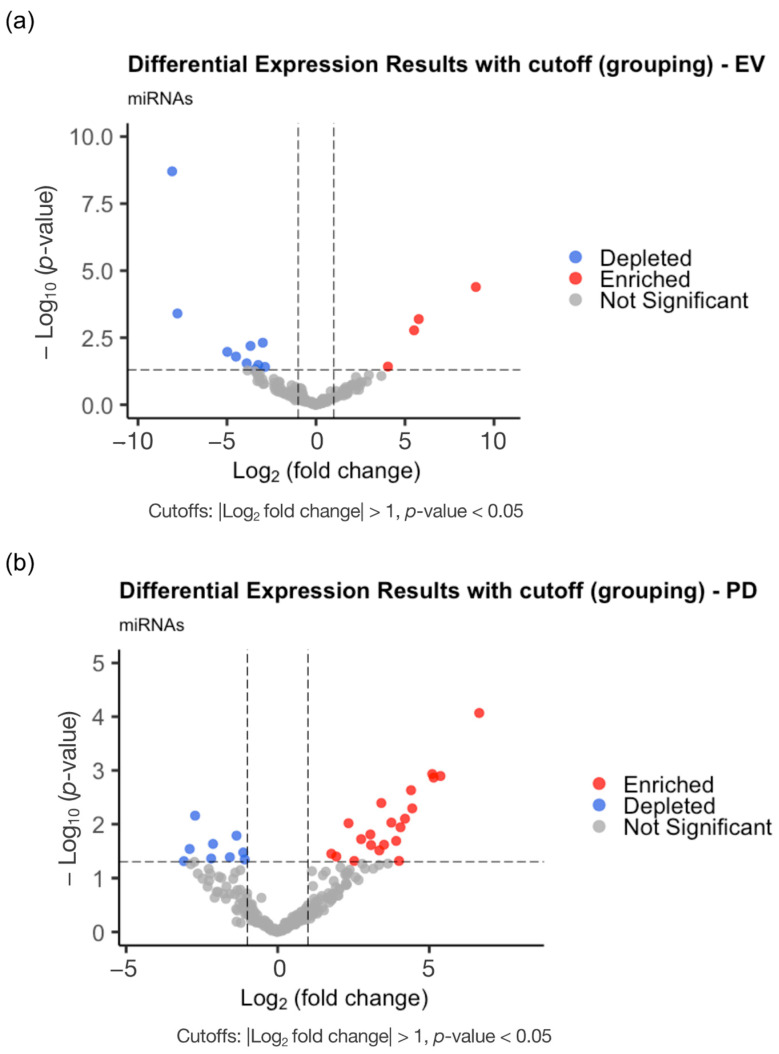
Volcano plots showing differences between healthy (*n* = 13) and cancer (*n* = 12) miRNAs in EVs (**a**) and in PD (**b**) populations. Fold-change (log2 FC) between healthy and patients with cancer with miRNA of more than 1-fold enrichment (red dots) or depletion (blue dots); Cutoffs (dash lines) correspond to |Log_2_ fold change| > 1 and *p*-value < 0.05.

The differences in the enrichment of specific miRNAs/isomiRs between the healthy (*n* = 13) and cancer samples (*n* = 12) were investigated in the EVs (Figure 5a) and in the PD (Figure 5b) populations. In the EV population (CD9-, CD63-, or CD81-positive), fourteen miRNAs/IsomiRs were described as differentially expressed, encompassing the enrichment of four miRNAs and the depletion of ten miRNAs (Figure 5a, Supplementary Table S1, Supplementary Figure S3a). Interestingly, the PD population (CD61-positive) exhibited a more complex profile, containing twenty-eight differently expressed miRNAs/IsomiRs, with nineteen miRNAs/IsomiRs enriched and nine miRNAs/IsomiRs depleted in the patients with NSCLC compared to those of the healthy controls (Figure 5b, Supplementary Table S2, Supplementary Figure S3b).

## 3. Discussion

In the present study, we have employed a prior optimized immunocapturing protocol to extract and analyze the molecular content of two extracellular vesicle populations directly from routinely harvested and stored plasma samples. Our method enables the collection of either the overall EV population, or a subpopulation of platelet-derived vesicles (PD). While some overlap between the two populations is likely (e.g., PD vesicles which are CD61-positive may also express common EV surface markers, such as CD9, CD63, and CD81), our analysis revealed distinct molecular enrichment profiles in the two isolates.

Although our analysis showed a similar distribution of RNA classes within the extracellular vesicles isolated from the healthy individuals and those with cancer (Figure 2a–d), with the biggest populations presented after the removal of rRNA, being microRNA and tRNA, in both the sample groups. These two types of RNA are often the most enriched RNA types in plasma vesicles due to their stability, roles in cell-to-cell communication, and ability to reflect cellular conditions [13]. The relative percentages of microRNA and tRNA within the vesicle subpopulations were inverted. The PD population demonstrated a higher abundance of microRNAs compared to that of the overall EV population, indicating the preferential enrichment of these regulatory molecules within the PD vesicles. While the opposite has been verified with the amount of tRNA. The EV population exhibit the greater enrichment of tRNA relative to the PD population (Figure 2d). This inverse distribution suggests distinct RNA sorting mechanisms and molecular profiles between these two EV subpopulations, reflecting potential differences in their biological functions and origins. We suggest that this difference may be due to the primary source of PD being activated platelets, which give priority to packaging miRNAs associated with inflammation, thrombosis, and intercellular signaling. On the other hand, the EV population, which comes from a variety of cellular sources, has the ability to specifically package tRNAs and other RNAs linked to metabolic regulation or stress.

In a previous study that used the same isolation method and the Nanostring nCounter platform to characterize the mRNA content of plasma vesicles from patients with lung cancer, the PD population showed higher mRNA counts and more DE mRNAs when compared to those of the overall plasma EVs [11]. These findings support the idea that PD vesicles may play a more active role in gene regulation and intercellular communication, particularly in the context of pathological processes such as lung cancer.

While both the EVs and the PD exhibit similar percentages of canonical and isoform miRNAs, the distinct trends in truncations, elongations, and non-templated additions (NTAs) highlight their unique contributions. These findings underscore the functional specialization of EVs and PDVs, with EVs potentially supporting systemic tumor progression, and PDVs modulating local tumor environments (Figure 4).

The EV population (CD9, CD63, or CD81) showed 14 differentially expressed miRNAs (4 enriched and 10 depleted) (Figure 5a, Supplementary Table S1, Supplementary Figure S3a). Interestingly, the PD population (CD61-positive) exhibited a more complex profile, with 28 differently expressed miRNAs (19 enriched and 9 depleted) in the patients with NSCLC compared to those of the healthy controls (Figure 5b, Supplementary Table S2, Supplementary Figure S3b). This gene expression analysis revealed that the PD population exhibits a more distinct and complex miRNA expression profile compared to that of the EV population, highlighting its potential as a more informative biomarker source for distinguishing patients with NSCLC from healthy controls. From the DE miRNAs/IsomiRs, some potential NSCLC biomarkers have been listed, such as the isoforms of miR-221-3p and miR-21-5p. These two miRNAs are frequently described as overexpressed in patients with NSCLC and their plasma-derived vesicles. The EV population showed the enrichment of hsa-miR-221-3p_nta_T (4.04 fold-change), and the PD population evidenced the enrichment of hsa-miR-21-5p_mv (1.77 fold-change). Elevated levels of these miRNAs are associated with tumor progression and poor clinical outcomes [14,15]. Another study suggested miR-221-3p and miR-21-5p obtained from plasma vesicles may serve as reliable predictors of the response to radiation treatment (Hassanin and Ramos 2024 [2]).

Interestingly, the isomiRs of mir-451a were depleted in both in the PD and EV populations, namely hsa-miR-451a_trunc (EV), hsa-miR-451a_trunc (PD), and hsa-miR-451a_mv (PD), (3.67, 2.13, and 2.72 fold-changes, respectively) (Figure 5a,b, Supplementary Tables S1 and S2). Mir-451a is commonly deregulated in NSCLC, and its expression is negatively associated with prognosis of patients with NSCLC [16]. The canonical form of hsa-miR-100-5p is enriched in the PD population (5.15 fold-change) of the samples analyzed in this study. All the samples used in this study derive from patients with stage IV NSCLC (Table 1), and hsa-miR-100-5p has been shown to be involved in drug resistance to Cisplatin. This observation makes this miRNA a very interesting candidate as a clinical biomarker for therapy selection and monitoring [17].

Although different surface markers were used to isolate the vesicle subpopulations, the possibility of marker overlap between the vesicle populations might complicate the specificity of the results. In future studies, the extent to which CD61 (PD) vesicles can also be CD9-, CD63-, or CD81-positive (EV) should be assessed. We expect the partial overlap of isolated subpopulations due to the magnetic beads used for immunocapture. The observed differences in this study, though promising, require further validation in larger and more diverse patient cohorts to enable the more comprehensive assessment of our findings and to explore the utility of such populations as a source of biomarkers for diagnostic and prognostic purposes in a clinical setting. Finally, the use of MACS beads for isolation demonstrates the feasibility of integrating plasma vesicle-based assays into clinical workflows, as magnetic beads are already widely used in automated systems and other diagnostic protocols. This compatibility facilitates standardization across multiple centers, enhancing the potential for clinical application.

## 4. Materials and Methods

Blood samples were collected from 13 healthy donors (H) and 12 patients with NSCLC (C) (Table 1). For each sample, 1 mL of plasma was isolated following the ThromboSeq protocol [12]. Two distinct populations of vesicles were then immunocaptured from plasma based on their membrane surface markers using magnetic beads: Magnetic Activated Cell Sorting (MACS) beads triple-conjugated with anti-CD9, CD63, and CD81 antibodies to immunocapture plasma total EVs (EVs); and MACS beads conjugated with anti-CD61 to immunocapture platelet MPs (Platelet Dust, PD). Following the isolation of the vesicles, total RNA was extracted. Library preparation and sequencing were performed using the NEXTFLEX^®^ Small RNA-Seq Kit (Bioo Scientific; cat no. NOVA-5132-05; Austin, TX, USA) (Figure 1).

**Table 1:** Information on patients with non-small-cell lung cancer (NSCLC) and healthy donors (n.a. not applicable).

	Age (Median)	Age (Min)	Age (Max)	Gender (Female)	Gender (Male)	Cancer Stage	Total
**Cancer (NSCLC)**	61	45	78	3	9	IV (12)	12
**Healthy**	62	50	86	4	9	n.a.	13

### 4.1. Plasma Isolation from Blood Samples

Blood samples were collected in a BD Vacutainer tube containing 6 mL of anticoagulant (EDTA) and stored at room temperature (RT) until centrifugation, which occurred within 8 h of collection. After centrifugation at 120× *g* for 20 min at RT, the blood samples show three phases: platelet-rich plasma (PRP), the leukocyte phase, and the erythrocyte phase (from upper to lower phases) (Figure 1). The PRP was carefully pipetted into a separate tube and centrifuged at 360× *g* for 20 min at RT. The supernatant was then transferred into another tube, leaving just the platelet pellet behind. This supernatant (plasma) was subsequently centrifuged at 4636× *g* for 10 min at RT. Finally, the resulting supernatant, corresponding to platelet-poor plasma (PPP), was pipetted into a 2 mL Eppendorf tube without disturbing the pellet and stored at −80 °C until further processing.

### 4.2. Extracellular Vesicles Isolation from Plasma

Vesicle isolation from plasma was performed via immunoprecipitation following a protocol optimized by Fortunato and colleagues [11]. This protocol involves the use of MACS beads (Miltenyi Biotec; Bergisch Gladbach, Germany) conjugated with antibodies that target surface markers specific to the vesicles populations (EVs and PD) being isolated and studied.

First, the beads were added to 500 μL of platelet free-plasma, previously aliquoted into two Eppendorf tubes. Two types of beads were used; 3 μL beads of MACS CD61 (Miltenyi Biotec; cat. no. 130-051-101) was added into one of the tubes to capture the platelet EVs (PD), and 1 μL beads of MACS triple-coated (CD9, CD63 and CD81) (Miltenyi Biotec; cat. no. 130-048-101) was added to the other tube to capture the triple-coated EVs (EVs). The tubes with beads were incubated for 1 h at RT with agitation, and later MACS columns (Miltenyi Biotec; cat. no. 130-042-701) were equilibrated with 100 μL of PBS-TX100 (Triton X-100, 1%) and washed with 500 μL of PBS. Next, immunoprecipitation reactions were loaded into pre-equilibrated MACS columns; they were washed 3 times with 20 μL of PBS-tween20 0.1% and 2 times with 400 μL of PBS. The beads were eluted in 100 μL PBS with plungers into a new labeled tube. The samples were processed on the same day for RNA extraction from the EVs (Figure 1).

### 4.3. RNA Isolation from Extracellular Vesicles (EV) and Platelet Dust (PD)

In order to isolate the RNA from EVs, the TRIzol LS reagent user guide was followed with some modifications. First, the EV and PD immunoprecipitated samples were brought to a volume of 250 μL with PBS and kept on ice. A total of 750 μL of TRIzol LS Reagent (Thermo Fisher Scientific; cat. no. 10296028; Waltham, MA, USA) was added into the samples (ratio TRIzol/sample was 3:1) and vortexed 30 s before being frozen at −80 °C for later processing. The samples were thawed, and 200 μL of chloroform was added; the tubes were shaken vigorously for 20 to 30 s and incubated for 5 min at RT. Then, the samples were centrifuged at 12,000× *g* for 15 min at 4 °C, and the aqueous phase was carefully transferred to a new tube, which contained the RNA (the lower phase, which was pink and contained the proteins, was stored at −80 °C). To precipitate the RNA, 2.5 μL of glycogen, and afterwards 500 μL of isopropanol were added to the aqueous phase. The samples were incubated for 1 h at −80 °C, and then they were thawed and centrifuged at 12,000× *g* for 10 min at 4 °C. The total RNA precipitate formed a white gel-like pellet at the bottom of the tube, and the supernatant was discarded carefully. After that, RNA washing was conducted by adding 1 mL of 75% ethanol, centrifuging at 7500× *g* for 5 min at 4 °C, and removing the supernatant as much as possible (RNA pellets were air-dried for 5–10 min). Finally, the pellets were resuspended in 10 μL of RNase-free water at RT, and the samples were stored at −80 °C (Figure 1).

### 4.4. Quality Control of the RNA Isolated (Bioanalyzer)

The quality check of the isolated RNA was conducted with Agilent RNA 600 Pico Kit (Agilent Technologies; cat. no. 5067-1513; Santa Clara, CA, USA) following the protocol provided by the company (G2938-90046 Rev. C).

### 4.5. Small RNA Sequencing

The small RNA library preparation of the total RNA samples was performed using the NEXTFLEX^®^ Small RNA-Seq Kit (Bioo Scientific; cat no. NOVA-5132-05; Austin, TX, USA) and following the protocol provided by the company. All the reagents needed were included in the kit.

The final PCR product was subjected to a size-selection step prior to sequencing. Size selection was performed using the PAGE gel electrophoresis (BluePippin; Sage Science; Beverly, MA, USA) of the PCR products to isolate the fragments within the range of interest. Fragments longer than 140 bp were preserved and sent for sequencing.

MiRNA libraries were sequenced on NovaSeq6000 (Illumina; San Diego, CA, USA). Paired-end (150 bp fragments) sequencing was conducted and with a total output from 10 to 40 million reads per sample, and FASTQ files were acquired (Figure 1).

### 4.6. Data Analysis

Pre-processing, including quality checks and adapter trimming, alignment, and quantification, were performed with the sRNAbench tool (v2.0) [18] (Supplementary Figure S2). Small non-coding RNAs (sncRNAs) with a low number of reads were filtered using the filterByExpr function from the EdgeR package [19] in R (version 4.1.2). The filterByExpr function was applied with default parameters to retain reads with more than 10 counts in a minimum number of samples, where the minimum number of samples was determined based on the smallest group size (12 samples) and all the “black and white” cases. Differential expression analysis was performed with the sRNAde tool [18], and the results from the EdgeR package [19] were selected for further exploration. The most significant differentially expressed small non-coding RNAs were identified using |log2FC| > 1 and *p*-value < 0.05 as thresholds.

### 4.7. Reference Databases

Expression profiling was performed using the reference libraries miRBase (v22) [20], GtRNAdb (v20) [21], and RNAcentral (v21) [22] for the detection of miRNAs, tRNAs, and all other types of small non-coding RNA (sncRNAs), respectively. Alignment was performed sequentially using Bowtie under sRNAbench, first detecting the miRNAs, followed by the tRNAs, and then all the other non-coding RNAs, allowing for only one mismatch (Supplementary Figures S1 and S2) (Figure 1).

### 4.8. miRNA/IsomiRs Analysis

In this study, the miRNAs were classified into two main groups: the canonical miRNAs and isomiRs. The isomiRs group is subdivided into two big subgroups composed of templated isoforms and non-templated isoforms (Supplementary Figure S1). The templated isoforms group includes all the miRNA sequence variants referring to the ability of aligning to canonical pre-miRNA sequences (precursor miRNAs refer to the hairpin precursors of miRNAs formed by the cleavage of primary miRNAs by DCGR8 and Drosha). The three subgroups of templated isoforms are as follows: (1) elongations: isomiRs with nucleotide additions on the 5′ end, the 3′ end, or on both ends; (2) truncations: isomiRs with deletions on the 5′ end, the 3′ end, or both ends; (3) multivariations: isomiRs with combination of modifications on the 5′ and 3′ ends and single-nucleotide polymorphisms (SNPs) (Supplementary Figures S1 and S2) (Figure 1). Regarding the non-templated isoforms, these miRNA sequence variants are characterized with nucleotide additions on the 3′ end that cannot be aligned to canonical pre-miRNA sequences [23,24,25]. The subgroups of non-templated isoforms are splitted in four different groups based on the last nucleotide. NTA-A: isomiRs sequence ending with A; NTA-U(T): isomiRs sequence ending with U; NTA-C: isomiRs sequence ending with C; and NTA-G: isomiRs sequence ending with G (Supplementary Figures S1 and S2) (Figure 1).

After analyzing and detecting the different isoforms using sRNAbench [18], the clustering analysis of miRNAs was conducted based on various isoform types as previously described in R. Differential expression analysis was also performed on the clustered data, following the same procedure outlined on Section 4.6 “Data Analysis” (Figures S1 and S2) (Figure 1).

## 5. Conclusions

Patients with lung cancer show distinct changes in extracellular vesicle RNAs, which correlate with tumor behavior, their response to therapy, and prognosis. This highlights their potential as cancer biomarkers [2,3] and the importance for a deeper investigation of the distribution of various RNA types across different plasma vesicle populations.

The distribution of different RNA categories between the cancer and healthy samples were comparable, with some variation observed in the tRNA and microRNA levels. We have shown that the PD (CD61+) vesicle population in plasma has unrevealed potential as a source of microRNAs, being more enriched in microRNA than the EV (CD9+, CD63+, or CD81+) population. This exploratory study advances our understanding of miRNA composition within different plasma vesicle populations, shedding light on the biology of plasma vesicles and their contents.

Importantly, the utilization of a novel extracellular vesicle isolation method based on MACS beads not only enables the specific enrichment of specific populations of plasma vesicles, but also demonstrates the feasibility of integrating plasma vesicle-based assays into clinical workflows [11]. Given that magnetic beads are already widely employed in automated platforms and diagnostic protocols, this compatibility facilitates standardization across multiple centers, thereby enhancing the translational potential of our approach in both preclinical and clinical studies.

Expanding our understanding of the molecular content of plasma and their extracellular vesicles, including Platelet-derived Extracellular Vesicles (“Platelet Dust”), the most abundant extracellular vesicles in plasma [1], and their role in disease, could pave the way for improved diagnostic and therapeutic strategies in clinical settings.

## Data Availability

The sequencing data generated and analyzed in this study will be available upon publication of this study in the NCBI GEO database under accession number GSE244416.

## Supplementary Materials

The following supporting information can be downloaded at: https://www.mdpi.com/article/10.3390/ncrna11030038/s1, Figure S1: miRNA groups and subgroups. Figure S2: Data processing and analysis pipeline. Table S1: DE miRNAs in EV (CD9+, CD63+ and CD81+) population. Fold change (log2 FC) between healthy and cancer patients with miRNA of more than 2-fold enrichment or depletion listed. Table S2: DE miRNAs in PD (CD61+) population. Fold change (log2 FC) between healthy and cancer patients with miRNA of more than 2-fold enrichment or depletion listed.

## Abbreviations

ATP	Adenosine Triphosphate
NSCLC	Non-small-cell Lung Cancer
cDNA	Complementary DNA
DNA	Deoxyribonucleic Acid
ELISA	Enzyme-Linked Immunosorbent Assay
EV(s)	Extracellular Vesicle(s)
MACS	Magnetic Activated Cell Sorting
miRNA	MicroRNA
mRNA	Messenger RNA
ncRNA	Non-Coding RNA
NSCLC	Non-Small-Cell Lung Cancer
PBS	Phosphate-Buffered Saline
PD	“Platelet Dust”, Platelet-derived particles/vesicles
PCR	Polymerase Chain Reaction
PPP	Platelet-poor plasma
PRP	Platelet-rich plasma
RNA	Ribonucleic Acid
RT	Room temperature
RT-qPCR	Reverse Transcription Quantitative Polymerase Chain Reaction
TEM	Transmission Electron Microscopy
