# Supplementary material for: Small RNA Landscape of Platelet Dust: Platelet-Derived Extracellular Vesicles from Patients with Non-Small-Cell Lung Cancer
Source: Noncoding RNA. 2025 May 7;11(3):38. doi: 10.3390/ncrna11030038 (PMC12101397; doi:10.3390/ncrna11030038)

Supplementary Figure S1. miRNA groups and subgroups

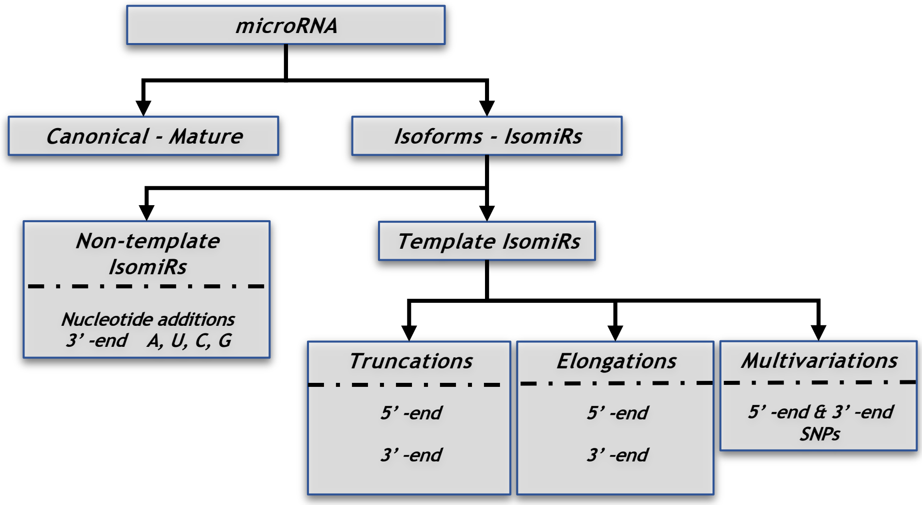

Supplementary Figure S2: Data processing and analysis pipeline

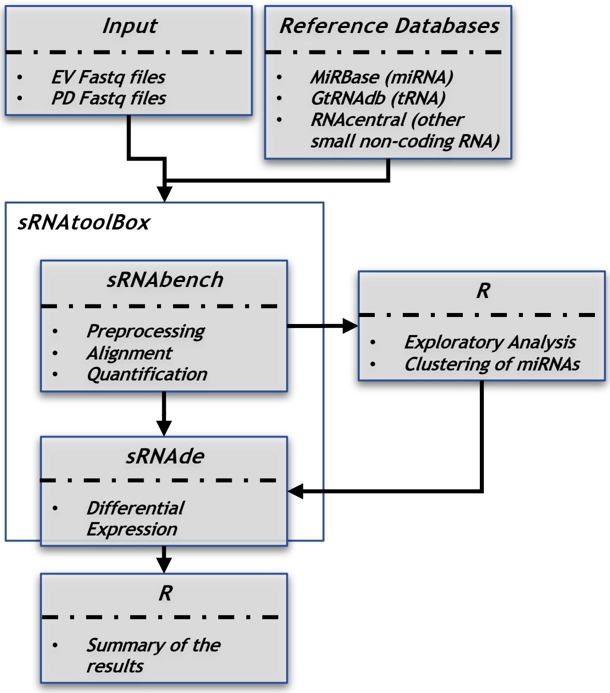

Supplementary Table S1. Differentially Expressed (DE) miRNAs in EV (CD9+, CD63+ and CD81+) population. Fold change (log2 FC) between healthy and cancer patients with miRNA of more than 2-fold enrichment or depletion listed.

Name	log2FoldChange	pvalue	-log10pvalue
hsa-miR-409-3p_elong	-7,783599901	0,00039460777	3,403834368
hsa-let-7d-5p_trunc	5,774015879	0,0006399665205	3,193842745
hsa-miR-151a-3p_nta_T	5,511228332	0,001677739746	2,775275407
hsa-miR-15b-5p_mv	-2,990743673	0,004858665484	2,313483001
hsa-miR-451a_trunc	-3,678519826	0,006407994203	2,19327789
hsa-let-7d-3p	-4,982294644	0,01064877004	1,972700551
hsa-miR-143-3p	-4,492919833	0,01602060181	1,795321174
hsa-let-7i-5p_trunc	-3,892362232	0,02854406517	1,544484176
hsa-miR-191-5p	-3,244044474	0,03291053043	1,482665118
hsa-miR-221-3p_nta_T	4,041318503	0,03770843204	1,423561526
hsa-miR-203a-3p_mv	-2,864626583	0,03877864507	1,411407369
hsa-miR-191-5p_mv	-3,410346339	0,04750495293	1,323261108
hsa-miR-423-5p_nta_A	-8,083833533	0,000000001982351868	8,702819256
hsa-miR-148a-3p	8,981216063	0,00004061920519	4,391268579

Supplementary Table S2. Differentially Expressed (DE) miRNAs in PD (CD61+) population. Fold change (log2 FC) between healthy and cancer patients with miRNA of more than 2-fold enrichment or depletion listed.

Name	log2FoldChange	pvalue	-log10pvalue
hsa-miR-222-3p_nta_A	5,098902329	0,001166514225	2,933109961
hsa-miR-28-3p_nta_A	5,37103499	0,001271078027	2,895827789
hsa-miR-100-5p	5,145914532	0,001361502324	2,865981613
hsa-let-7g-5p_elong	4,399365561	0,002335289598	2,631659255
hsa-let-7c-5p	3,418283083	0,004040354026	2,393580579
hsa-miR-191-5p_elong	4,43795407	0,00508767581	2,29348057
hsa-miR-451a_mv	-2,724750954	0,006942720425	2,158470323
hsa-miR-197-3p	4,200683956	0,007924220551	2,101043445
hsa-miR-148b-3p	3,751533106	0,009362019663	2,028630451
hsa-miR-26a-5p_trunc	2,337083274	0,009594850066	2,017961807
hsa-miR-193b-3p_trunc	4,051793684	0,01143323179	1,941830992
hsa-miR-100-5p_trunc	3,059381093	0,01552583833	1,808944941
hsa-miR-16-5p	-1,357181098	0,01634177042	1,786700895
hsa-let-7b-5p_mv	2,752960081	0,01898866182	1,72150564
hsa-miR-4433b-3p	3,908893673	0,02047179996	1,688843971
hsa-miR-451a_trunc	-2,134665547	0,02329461348	1,632744491
hsa-miR-146b-5p_elong	3,508684199	0,02411259839	1,617755987
hsa-miR-28-5p	3,080398717	0,02446915751	1,611380983
hsa-miR-423-5p_nta_A	-2,904428982	0,02891680881	1,538849636
hsa-miR-26a-5p_elong	3,353078338	0,0307463558	1,512206351
hsa-let-7a-5p_trunc	-1,135733202	0,03356889531	1,47406295
hsa-miR-21-5p_mv	1,768830743	0,0354343615	1,450575389
hsa-miR-26a-5p_mv	1,934386401	0,03997037078	1,398261823
hsa-miR-486-5p_trunc	-1,577322293	0,04116706652	1,385450079
hsa-miR-4486_trunc	-2,195150958	0,04354831637	1,361028631
hsa-miR-92a-3p	-1,087544179	0,04536090971	1,343318244
hsa-miR-1260a_trunc	4,003378464	0,04821811429	1,316789778
hsa-miR-151a-5p_trunc	2,525790204	0,0483501716	1,31560198
hsa-miR-320a-3p_trunc	-3,094199487	0,04878558676	1,311708467
hsa-miR-1-3p_nta_G	6,649837573	0,00008560627127	4,067494419

Supplementary Figure S3. Reads per million (RPM) of the miRNAs enriched and depleted in the EV (CD9+, CD63+ and CD81+) population (a) and in the PD (CD61+) population (b).

(a) miRNAs (RPM) - EV

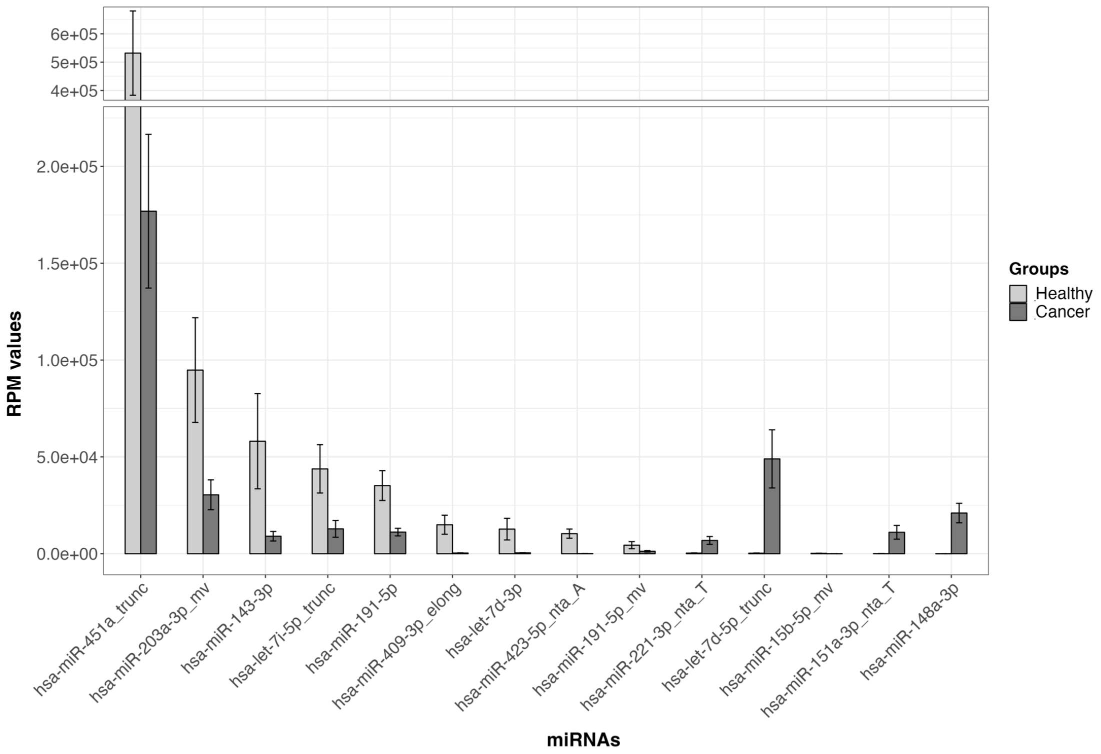

(b) miRNAs (RPM) - PD

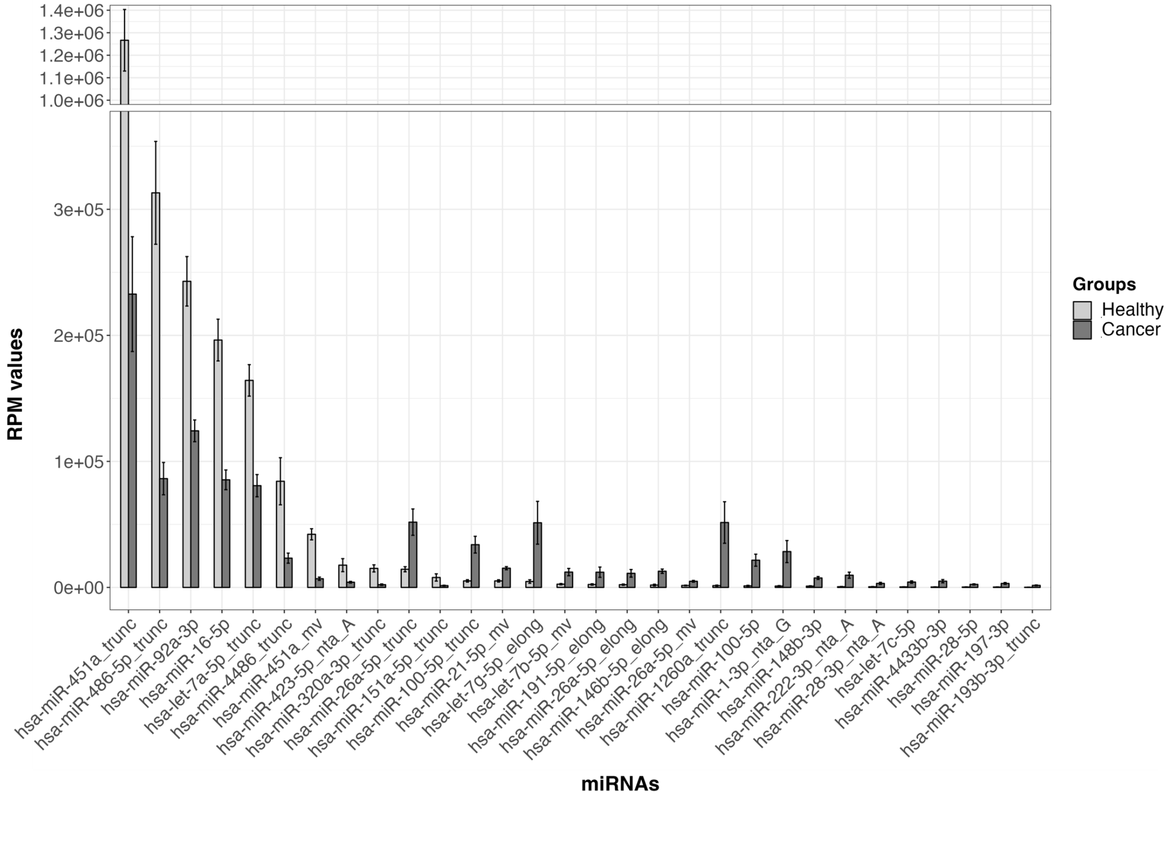

Supplement: Supplementary file 1 [file ncrna-11-00038-s001.zip › ncrna-3478745-supplementary.pdf]
